# Polarized electron beams elastically scattered by atoms as a tool for testing fundamental predictions of quantum mechanics

**DOI:** 10.1038/s41598-018-23660-4

**Published:** 2018-03-29

**Authors:** Maurizio Dapor

**Affiliations:** 1 European Centre for Theoretical Studies in Nuclear Physics and Related Areas (ECT*-FBK), Trento, 38123 Italy; 2 Trento Institute for Fundamental Physics and Applications (TIFPA-INFN), Trento, 38123 Italy

## Abstract

Quantum information theory deals with quantum noise in order to protect physical quantum bits (*qubits*) from its effects. A single electron is an emblematic example of a *qubit*, and today it is possible to experimentally produce polarized ensembles of electrons. In this paper, the theory of the polarization of electron beams elastically scattered by atoms is briefly summarized. Then the POLARe program suite, a set of computer programs aimed at the calculation of the spin-polarization parameters of electron beams elastically interacting with atomic targets, is described. Selected results of the program concerning Ar, Kr, and Xe atoms are presented together with the comparison with experimental data about the Sherman function for low kinetic energy of the incident electrons (1.5eV–350eV). It is demonstrated that the quantum-relativistic theory of the polarization of electron beams elastically scattered by atoms is in good agreement with experimental data down to energies smaller than a few eV.

## Introduction

Polarization phenomena are of huge technological and theoretical interest. Today it is possible to experimentally produce polarized ensembles, and the case of spin-polarized beams deserves particular attention for its simplicity, for its potential technological applications, and for its conceptual aspects. Indeed, an electron is an emblematic example of a physical quantum bit, or *qubit*^1,2^, as its spin has only two possible levels. Also the polarization of a photon and the states of an atom (ground-state and excited state) represent examples of *qubits*. Quantum noise (which is due to nature itself and not to our lack of information) affects *qubits*. It is therefore important to be able to model and control quantum noise. The aim of the modern quantum information theory, for example, is to create methods to properly deal with quantum noise in order to protect *qubits* from its effects^2^.

The study of electron beams interacting with atoms has many applications and it is, in particular, very important for the Monte Carlo simulation of the transport of electrons in solid targets^3–6^. In particular, bremsstrahlung resulting from polarized electrons has been investigated using the Monte Carlo method in order to model of the polarization transfer from electrons to photons^7^ (BEPSI code).

Furthermore, the comparison between theoretical and experimental investigations about spin-polarized electron beams represents a very simple test for the fundamental predictions of quantum mechanics.

This paper deals with the study of spin-polarized electron beams produced by scattering with atoms. An electron beam is a quantum system in a mixed state of spin orientations. A beam of electrons is polarized when the electron spins have a preferential orientation^8^. In other words, in a polarized electron beam the two populations of spin orientations are different. A similar situation occurs in ferromagnetic materials, where the up- and down-spin electron densities of states are not the same^9,10^. On the one hand, the spins of electrons produced by thermal emission have arbitrary directions. On the other hand, today it is experimentally possible to produce electron beams with the two possible spin orientations not equally populated.

If all the spins have the same orientation, the beam is said to be *totally polarized*. A beam of electrons can also be *partially polarized* when the majority of the spins have the same orientation. When the two possible spin orientations are equally populated, then the beam is said to be *unpolarized*^8^.

Within the density-matrix formalism a set of observable quantities can be defined^8^. These observables–the polarization parameters *S*, *T*, and *U* (see Section 2 for their definitions and details of calculations), which depends on the scattering angle and on the incident electron kinetic energy–describe the elastic scattering process, providing a way for calculating the differential elastic scattering cross-section for spin-polarized electron beams. In fact, while for an unpolarized electron beam the differential elastic scattering cross-section can be determined without any knowledge of the *S*, *T*, and *U* spin-polarization parameters, for the case of polarized electron beams the cross-section depends on the *S* function (also known as the Sherman function, or the left-right asymmetry function) and on the state of polarization of the beam. Furthermore it is possible to demonstrate that, after an elastic collision, an initially unpolarized electron beam acquires a polarization (whose magnitude is equal to the Sherman function *S*). In general, for an initially polarized beam, the final polarization after an elastic scattering collision is a function of all the *S*, *T*, and *U* spin-polarization parameters. The determination of the spin-polarization parameters in the elastic scattering of electrons from atomic targets has been investigated both experimentally and theoretically by many investigators^11–43^. Note that measurement of spin-polarization can be made by using Mott polarimetry, that has been demonstrated by Sromicki *et al*. to be applicable up to 14 MeV^35^. Theoretical calculations about 3 MeV electrons has been recently proposed by Wlodarczyk *et al*.^43^. High energy does not immediately concerns quantum information, so that this paper is mainly interested in describing spin-polarization phenomena in the low energy regime. Also note that the code system ELSEPA (Elastic Scattering of Electrons and Positrons by Atoms)^40^ provides the scattering amplitudes (both direct and spin-flip) with the aim of calculating the differential, total, and transport elastic scattering cross-sections of Dirac particles (electrons, positrons) elastically interacting with atoms, positive ions, and molecules. From the calculated scattering amplitudes it is possible to easily determine the polarization parameters. On the other hand ELSEPA does not allow to made calculations down to a few eV: ELSEPA authors consider questionable the reliability of the results of the calculation when the electron energy is smaller than 100 eV. As a matter of fact, ELSEPA does not allow to made calculations when the electron energies are smaller than 10 eV.

The calculation of the *S*, *T*, and *U* spin-polarization parameters is crucial for the complete knowledge of the elastic scattering processes. This paper describes a program suite (named POLARe), based on the numerical solution of the Dirac equation in a central field and written for the calculation of the spin-polarization parameters of electron beams with low energy, down to 1.5 eV.

The POLARe program suite is presented together with selected results about elastic scattering collisions of electrons with Ar, Kr, and Xe atoms. Comparisons with both experimental data and ELSEPA results are also provided, in order to investigate the accuracy of the described methods for very low and intermediate electron kinetic energies.

## Theoretical Remarks

The POLARe program suite is based on the Mott’s theory, that will be briefly summarized in the present section. Many details about the derivation of the formulas can be found in the Mott’s original paper^11^, and in the papers by the Lin *et al*.^13^, and by Bunyan and Schonfelder^14^. Concerning the equations describing the single- and double- scattering experiments, the reader can refer to the Kessler’s book^8^.

The relativistic partial wave expansion method (Mott’s theory)^11^ allows one to calculate the differential elastic scattering cross-section of electrons and positrons interacting with atomic targets. The Dirac’s equations for an electron (or a positron) in a central field may be written as

1 $$[W-V(r)+\mathrm{1]}{F}_{l}^{\pm }+\frac{d{G}_{l}^{\pm }}{dr}+\frac{1+{k}^{\pm }}{r}{G}_{l}^{\pm }=\mathrm{0,}$$

2 $$-[W-V(r)-\mathrm{1]}{G}_{l}^{\pm }+\frac{d{F}_{l}^{\pm }}{dr}+\frac{1-{k}^{\pm }}{r}{F}_{l}^{\pm }=\mathrm{0,}$$

where *W* is the particle energy expressed in units of *mc*^2^, *V*(*r*) is the atomic potential energy expressed in units of *mc*^2^ as well, *r* is the distance from the atom expressed in $\hslash /mc$ units, *m* is the electron (or positron) mass, *c* is the speed of the light, $\hslash =h\mathrm{/2}\pi$, and *h* is the Planck’s constant. The ± signs refer to the spin: + applies to spin up *i.e. j* = *l* + 1/2 while − corresponds to spin down *i.e. j* = *l* − 1/2. *k*^+^ = −(*l* + 1) while *k*^−^ = *l*. ${G}_{l}^{\pm }$ and ${F}_{l}^{\pm }$ are two functions of *r*. The following transformations

3 $${G}_{l}^{\pm }={A}_{l}^{\pm }\frac{\cos \,{{\varphi }}_{l}^{\pm }(r)}{r}$$

4 $${F}_{l}^{\pm }={A}_{l}^{\pm }\frac{\sin \,{{\varphi }}_{l}^{\pm }(r)}{r}$$

reduce the problem to the solution of the following first-order differential equation:

5 $$\frac{d{{\varphi }}_{l}^{\pm }(r)}{dr}=\frac{{k}^{\pm }}{r}\,\sin \,\mathrm{[2}{{\varphi }}_{l}^{\pm }(r)]-\,\cos \,\mathrm{[2}{{\varphi }}_{l}^{\pm }(r)]+W-V(r\mathrm{)}.$$

If we define

6 $${\tilde{{\varphi }}}_{l}^{\pm }=\mathop{\mathrm{lim}}\limits_{r\to \infty }{{\varphi }}_{l}^{\pm },$$

the phase shifts ${\delta }_{l}^{\pm }$ of the scattered waves in an elastic scattering experiment may be calculated by

7 $$\tan \,{\delta }_{l}^{\pm }=\frac{K{j}_{l+1}(Kr)-{j}_{l}(Kr)[(W+\mathrm{1)}\,\tan \,{\tilde{{\varphi }}}_{l}^{\pm }+\mathrm{(1}+l+{k}^{\pm })/r]}{K{n}_{l+1}(Kr)-{n}_{l}(Kr)[(W+\mathrm{1)}\,\tan \,{\tilde{{\varphi }}}_{l}^{\pm }+\mathrm{(1}+l+{k}^{\pm })/r]}$$

where

8 $${K}^{2}={W}^{2}-\mathrm{1,}$$

*j*_*l*_ are the regular spherical Bessel’s functions and *n*_*l*_ are the irregular spherical Bessel’s function (Neumann functions). If we indicate with *P*_*l*_(*x*) the Legendre’s polynomials and

9 $${P}_{l}^{1}(x)=\sqrt{1-{x}^{2}}\frac{d{P}_{l}(x)}{dx},$$

once the phase shifts are known, the direct and spin-flip scattering amplitudes [*f*(*θ*) and *g*(*θ*) respectively, where *θ* represents the scattering angle] are given by^11^

10 $$f(\theta )=\frac{1}{2iK}\sum _{l=0}^{\infty }\{(l+\mathrm{1)}\,[\exp \mathrm{(2}i{\delta }_{l}^{+})-\mathrm{1]}+l\,[\exp \mathrm{(2}i{\delta }_{l}^{-})-\mathrm{1]\}}{P}_{l}(\cos \,\theta )$$

11 $$g(\theta )=\frac{1}{2iK}\sum _{l=1}^{\infty }[\,-\,\exp \mathrm{(2}i{\delta }_{l}^{+})+\exp \mathrm{(2}i{\delta }_{l}^{-}]{P}_{l}^{1}(\cos \,\theta \mathrm{)}.$$

This is the relativistic partial wave expansion method.

Once the scattering amplitudes are known, it is possible to calculate the differential elastic scattering cross section as

12 $$\frac{d\sigma }{d{\rm{\Omega }}}=[|f(\theta {)|}^{2}+|g(\theta {)|}^{2}\mathrm{][1}+S(\theta ){{\bf{P}}}_{i}\cdot \hat{{\bf{n}}}]$$

where

13 $$\hat{{\bf{n}}}=\frac{{{\bf{k}}}_{i}\times {{\bf{k}}}_{f}}{|{{\bf{k}}}_{i}\times {{\bf{k}}}_{f}|}$$

and **k**_*i*_ and **k**_*f*_ are, respectively, the initial and final momenta of the electron (positron). In other words $\hat{{\bf{n}}}$ is the unit vector normal to the scattering plane. Let us indicate with ***s*** the spin operator for the spin 1/2 particles. Its components are the 2 × 2 Pauli matrices divided by two. Concerning **P**_*i*_, it represents the initial polarization vector. The polarization vector **P** is the mean value of 2**s** calculated over the functions of spin:

14 $${\bf{P}}=\langle 2{\bf{s}}\rangle $$

Regarding *S*(*θ*) it is a real function known as the Sherman’s asymmetry function. It is given by

15 $$S(\theta )=i\frac{f(\theta ){g}^{\ast }(\theta )-{f}^{\ast }(\theta )g(\theta )}{|f(\theta {)|}^{2}+|g(\theta {)|}^{2}}.$$

Since an unpolarized electron (positron) beam is composed of equal numbers of particles polarized parallel and antiparallel to a given direction (for example the incidence direction), averaging over the initial spin orientations we obtain

16 $${(\frac{d\sigma }{d{\rm{\Omega }}})}_{unpolarized}=\,|f(\theta {)|}^{2}+|g(\theta {)|}^{2}$$

and hence we can write that, in general,

17 $$\frac{d\sigma }{d{\rm{\Omega }}}={(\frac{d\sigma }{d{\rm{\Omega }}})}_{unpolarized}+i[f(\theta ){g}^{\ast }(\theta )-{f}^{\ast }(\theta )g(\theta )]{{\bf{P}}}_{i}\cdot \hat{{\bf{n}}}.$$

An interesting results concerning the initially unpolarized electron (positron) beams (**P**_*i*_ = 0) is that, after scattering, the final polarization **P**_*f*_ is a function of the angle of scattering *θ* and is given by^8^

18 $${{\bf{P}}}_{f}=S(\theta )\hat{{\bf{n}}}.$$

In words an electron (positron) beam initially not polarized, *i.e* composed of equal numbers of particles polarized parallel and antiparallel to the incidence direction (density matrix $\hat{\mu }$, *P* = 0, see Supplementary information), due to the scattering become polarized (density matrix $\hat{\rho }$, *P* ≠ 0, see Supplementary inforamtion). The magnitude of the polarization is the Sherman’s asymmetry function (sometimes called, for this reason, polarization function) and the direction is normal to the plane of scattering. The experimental evaluation of the asymmetry function is typically performed by the so called double scattering experiments^8^. Let be **k**_*f*1_ and **k**_*f*2_, respectively, the final momenta after the first and the second scattering. We have two scattering planes and the unit vectors normal to the two planes are

19 $${\hat{{\bf{n}}}}_{1}=\frac{{{\bf{k}}}_{i}\times {{\bf{k}}}_{f1}}{|{{\bf{k}}}_{i}\times {{\bf{k}}}_{f1}|},$$

20 $${\hat{{\bf{n}}}}_{2}=\frac{{{\bf{k}}}_{f1}\times {{\bf{k}}}_{f2}}{|{{\bf{k}}}_{f1}\times {{\bf{k}}}_{f2}|}$$

where, as before, **k**_*i*_ is the initial momentum. The differential elastic scattering cross section for the second scattering, if the beam is initially unpolarized, is given by

21 $$\frac{d{\sigma }_{2}}{d{{\rm{\Omega }}}_{2}}=[|f({\theta }_{2}{)|}^{2}+|g({\theta }_{2}{)|}^{2}\mathrm{][1}+S({\theta }_{1})S({\theta }_{2}){\hat{{\bf{n}}}}_{1}\cdot {\hat{{\bf{n}}}}_{2}]$$

where *θ*_1_ and *θ*_2_ are, respectively, the first and the second scattering angle. Then the cross section depends on the scattering angles, the Sherman functions and the angle between the scattering planes. Considering only the scatterings occuring in the same plane we have ${\hat{{\bf{n}}}}_{1}$ ⋅ ${\hat{{\bf{n}}}}_{2}$ = ±1. Let us define

22 $${\eta }_{l}\equiv {(\frac{d{\sigma }_{2}}{d{{\rm{\Omega }}}_{2}})}_{left},$$

23 $${\eta }_{r}\equiv {(\frac{d{\sigma }_{2}}{d{{\rm{\Omega }}}_{2}})}_{right}.$$

In practice when the two scatterings occur in the same plane, *η*_*l*_ and *η*_*r*_ are the differential elastic scattering cross sections for the second scattering corresponding, respectively, to ${\hat{{\bf{n}}}}_{1}$ ⋅ ${\hat{{\bf{n}}}}_{2}$ = +1 and ${\hat{{\bf{n}}}}_{1}$ ⋅ ${\hat{{\bf{n}}}}_{2}$ = −1. It is easy to see that

24 $$\varepsilon \equiv \frac{{\eta }_{l}-{\eta }_{r}}{{\eta }_{l}+{\eta }_{r}}=S({\theta }_{1})S({\theta }_{2}\mathrm{)}.$$

As a consequence a measure of *ε* for ${\theta }_{1}={\theta }_{2}=\bar{\theta }$ allows one to obtian ${S}^{2}(\bar{\theta })$. Once $|S(\bar{\theta })|$ is known for a given angle $\bar{\theta }$, a second experiment is performed varying *θ*_1_ and keeping constant ${\theta }_{2}=\bar{\theta }$. Since $|S(\bar{\theta })|$ is known from the first experiment, by utilizing the equation defining *ε* it is now possible to determine |*S*(*θ*_1_)| for different angles *θ*_1_.

In conclusion we note that, in the general case for which the initial polarization is not zero, *i.e*. for 0 ≤ |**P**_*i*_| ≤ 1, it is possible to show that^8^

25 $${{\bf{P}}}_{f}=\frac{[{{\bf{P}}}_{i}\cdot \hat{{\bf{n}}}+S(\theta )]\hat{{\bf{n}}}+T(\theta )[{{\bf{P}}}_{i}-({{\bf{P}}}_{i}\cdot \hat{{\bf{n}}})\hat{{\bf{n}}}]+U(\theta )\hat{{\bf{n}}}\times {{\bf{P}}}_{i}}{1+{{\bf{P}}}_{i}\cdot \hat{{\bf{n}}}S(\theta )}$$

where

26 $$T(\theta )=\frac{|f(\theta {)|}^{2}-|g(\theta {)|}^{2}}{{(d\sigma /d{\rm{\Omega }})}_{unpolarized}}$$

and

27 $$U(\theta )=\frac{f(\theta ){g}^{\ast }(\theta )+{f}^{\ast }(\theta )g(\theta )}{{(d\sigma /d{\rm{\Omega }})}_{unpolarized}}$$

Note that

28 $${S}^{2}(\theta )+{T}^{2}(\theta )+{U}^{2}(\theta )=1$$

The experimental determination of *T*(*θ*) and *U*(*θ*) is performed by triple scattering experiments^8^.

## The POLARe Program Suite Structure

**Figure 1:**
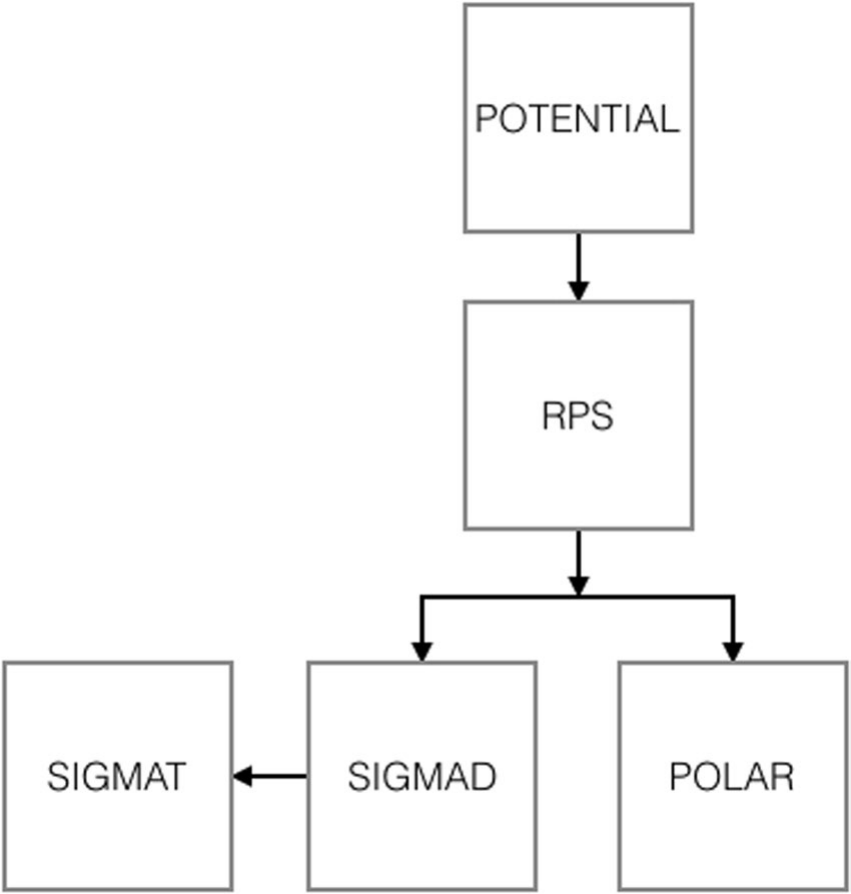
Block diagram of the POLARe program suite.

The POLARe program suite consists of five c routines set up for calculating the observables of interest for the complete description of the elastic scattering of electrons with atoms (see the block diagram in Fig. 1). In particular, a routine is aimed at computing the static atomic potential energy (POTENTIAL), another at computing the relativistic phase shifts (RPS), another at computing the *S*, *T*, and *U* polarization parameters (POLAR), another at computing the differential elastic scattering cross-section (SIGMAD), and finally another at computing the total elastic scattering cross-section and the first and the second transport elastic scattering cross-sections (SIGMAT). The POLARe program suite is a user friendly code freely obtainable on request to the author.

### Description of the Program Suite

The atomic potential energy is numerically assessed by the routine POTENTIAL in a radial grid (with origin in the nucleus) used as an input file by the program RPS, that calculates the phase shifts. In this work the atomic potential energy is taken from Cox and Bonham^15^. Since actually the only input required by the RPS routine is a set of (*r*, *V*) data, where *r* is the radial distance from the centre of the atomic nucleus and *V* is the atomic potential energy, the program POTENTIAL can be skipped. The numerical table including the potential can be obtained from other sources. For example, the Dirac-Hartree-Fock potential energy can be calculated ab-initio. In fact the modularity of the program suite allows to calculate the relativistic phase shifts, the differential elastic scattering cross sections, and the polarization parameters without any use of the POTENTIAL routine, if a numerical data-base of the potential energy is available. The POTENTIAL code can be, on the other hand, also used for the calculation of the potential energy using other best fits of the self consistent fields proposed in the literature such as, for example, the one given by Salvat *et al*.^44^. In fact the Salvat *et al*. potential has the same analytical form of the Cox and Bonham one, i.e. it is a superposition of a given number of Yukawa potentials (being the best fit of the previously calculated Dirac-Hartree-Fock-Slater potential). The program includes the set of potential parameters from *Z* = 1 to *Z* = 54 (Cox and Bonham potential^15^) and from *Z* = 1 to *Z* = 92 (Salvat *et al*. potential^44^). The sensitivity of the calculated data on the spacing of the grid of radii where the potential is tabulated is an important basic aspect, and it has been carefully considered. For the current calculations, the data spacing of the grid of radii was set to be equal to 2 × 10^−6^ nm.

The RPS program utilizes the previously calculated potential energy for numerically evaluating the phase shifts, according to the Mott theory briefly described in the previous section, using the fourth-order Runge-Kutta algorithm. The phase shifts are calculated from *l* = 0 to a maximum *l*, *l*_max_. The value of *l*_max_ is very important in order to obtain accurate results, and a reasonable criterium for its evaluation is then necessary. According to Salvat *et al*.^28^ it can be established, for example, looking for the convergence (to a desired accuracy) of the series describing the total and the first transport cross sections. The ELSEPA code system, on the other hand, calculates all the phase shifts with moduli larger than 10^−10 ^^41^. The RPS code estimates the value of *l*_max_ as ${l}_{{\rm{\max }}}\sim K\,{r}_{{\rm{\max }}}$, where *r*_max_ is the radius beyond which the potential energy becomes negligible (${r}_{{\rm{\max }}}\sim 2{\AA }-3{\AA }$, depending on the atomic number of the target)^45^. However, according to Koonin and Meredith^45^, this estimate is slightly low. Then the RPS code takes *l*_max_ = 2*K r*_max_. The special functions of the mathematical physics, used for the calculations of the phase shifts (Legendre polynomials *P*_*l*_, Bessel functions *j*_*l*_, Neumann functions *n*_*l*_) are calculated using recursion formulas, according to Abramowitz and Stegun^46^. See below for details about the calculation of the special functions of mathematica physics. For further numerical details about the RPS routine, see refs^31,38,47^. In the quoted references, it was demonstrated that the program works correctly in the interval of energies 350–1500 eV. On the other hand, the program was never validated for the evaluation of the Sherman function in the energy range 1.5–350 eV.

Once the phase shifts are known, they represent the input data for the POLAR and the SIGMAD codes, which calculate the *S*, *T*, and *U* parameters (POLAR) and the differential elastic scattering cross-section (SIGMAD) according to the theory depicted in Section 2. A further routine, named SIGMAT, can be used for calculating, by using the differential elastic scattering cross-sections provided by SIGMAD as input data, the total elastic scattering cross-section, and the first and the second transport elastic scattering cross-sections. SIGMAT uses the Bode’s quadrature formula for integrating the differential elastic scattering cross-section.

### Special Functions of Mathematical Physics

POLARe reserves a particular attention to the accurate calculation of the special functions of mathematical physics (Legendre polynomials *P*_*l*_, Bessel functions *j*_*l*_, Neumann functions *n*_*l*_). They are obtained using recursion formulas. In particular, the Legendre polynomials are calculated by using the following equation^46^:

29 $$(l+\mathrm{1)}{P}_{l+1}(u)+l{P}_{l-1}(u)=\mathrm{(2}l+\mathrm{1)}u{P}_{l}(u)\,.$$

A forward recursion in *l* allows to obtain *P*_*l*_ for any value of *l* starting from the explicit values *P*_0_(*u*) = 1 and *P*_1_(*u*) = *u*.

If we indicate by *f*_*l*_ any linear combination of the Bessel and Neumann functions (*f*_*l*_ = *aj*_*l*_ + *bn*_*l*_), where *a* and *b* are arbitrary coefficients, we have^46^:

30 $$x{f}_{l-1}-\mathrm{(2}l+\mathrm{1)}{f}_{l}+x{f}_{l+1}=0\,.$$

While the Neumann functions (*a* = 0, *b* = 1) can also be calculated using a forward recursion procedure (starting from the known functions *n*_0_(*u*) = −cos *u*/*u* and *n*_1_(*u*) = −cos *u*/*u*^2^ − sin *u*/*u*), the calculation of the Bessel functions (*a* = 1, *b* = 0) obtained using such a procedure introduces gross errors as *l* increases. POLAREe utilizes instead a backward recursion procedure, which provides very accurate results^45^. In the present version of the code, we start the backward procedure for the calculation of the Bessel functions with *j*_232_(*u*) = 0 and *j*_231_(*u*) = 9.9999 × 10^−300^, an arbitrarily small number, and then recur backwards to *l* = 0. The sequence obtained in such a way reproduces the Bessel functions to within an arbitrary normalization. The sequence of numbers obtained by the described backward procedure is then normalized so that *j*_0_(*u*) = sin *u*/*u*.

## Selected Results

The theory described in the Section 2 was implemented in the POLARe program suite which can perform, in particular, the calculation of all the polarization parameters *S*, *T*, and *U*.

We will first show the results of the calculation of the radial electron density for Ar, Kr, and Xe obtained using two popular screening functions, one proposed by Cox and Bonham^15^ and the other one by Salvat *et al*.^44^. The knowledge of the atomic potential energy is necessary to solve the Dirac equation and to calculate the electron radial density, using the Poisson’s equation.

Then we will present the calculations, performed using the POLARe code, of the Xe polarization parameters *S*, *T*, and *U* as a function of the scattering angle and of the incident electron kinetic energy. We will compare the calculations of the *S* function of Xe with the ELSEPA predictions. In the end the POLARe results will be compared with the Schackert^16^, Beerlage *et al*.^21^, Berger and Kessler^25^ and Dümmler *et al*.^29^ experimental data of Ar, Kr, and Xe.

### Calculation of the Electrostatic Atomic Potential and of the Electronic Density

The electrostatic atomic potential energy can be calculated as the product of the Coulomb potential energy multiplied by a screening function *ψ*(*r*), expressed as a superposition of Yukawa functions. The screening function is given by

31 $$\psi (r)=\sum _{i=1}^{p}\,{\gamma }_{i}\,\exp (\,-\,{\lambda }_{i}\,r\mathrm{)}.$$

The values of the parameters *p*, *γ*_*i*_ and *λ*_*i*_ can be established by looking for the best fit of the electrostatic atomic potential previously calculated using the Hartree-Fock method. POLARe can use the values of the parameters *p*, *γ*_*i*_ and *λ*_*i*_ provided by Cox and Bonham^15^ (best fit of Hartree-Fock calculations) and by Salvat *et al*.^44^ (best fit of Dirac-Hartree-Fock-Slater calculations). The electronic density can also be easily calculated, using the Poisson’s equation, as

32 $$\rho (r)=\frac{Z}{4\,\pi r}\sum _{i=1}^{p}\,{\gamma }_{i}\,{\lambda }_{i}^{2}\,\exp (\,-\,{\lambda }_{i}\,r\mathrm{)}.$$

**Figure 2:**
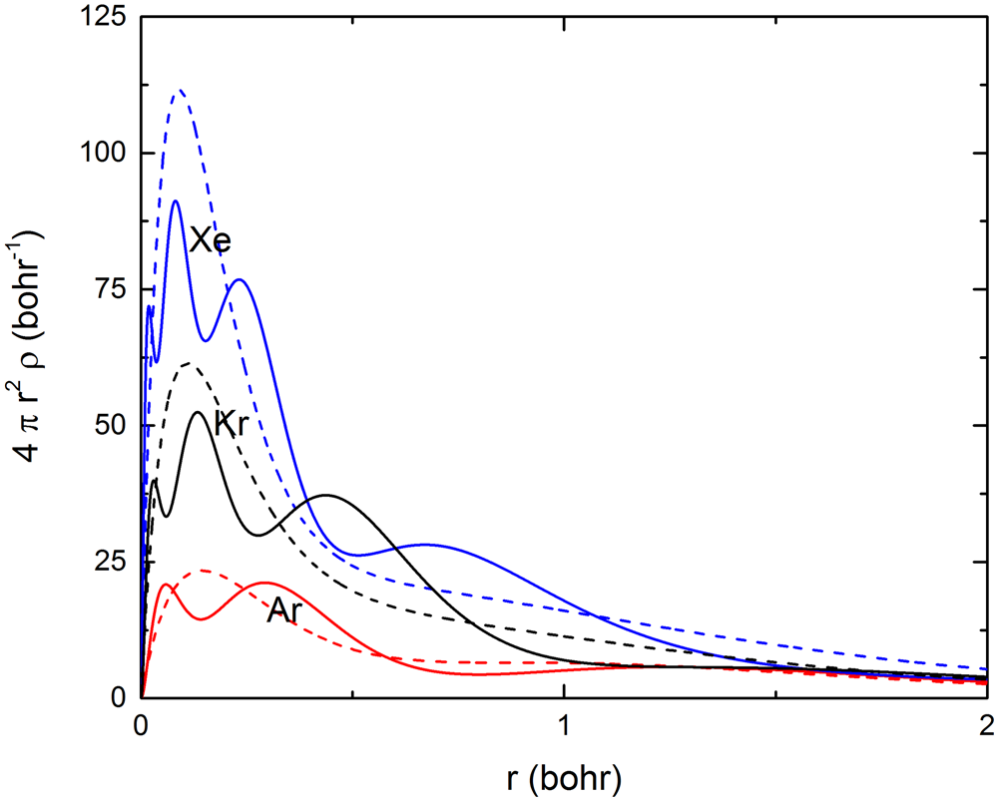
Radial density for Ar, Kr, and Xe. Solid lines: Cox and Bonham screening function^15^. Dashed lines: Salvat *et al*. screening function^44^.

In Fig. 2 we present the radial density for Ar, Kr, and Xe obtained using the Cox and Bonham and the Salvat *et al*. screening functions. The screening function of Cox and Bonham provides a rather accurate description of the details of the electronic radial density, while that of Salvat *et al*. gives an average of them.

### Calculation of *S*, *T*, and *U* as a Function of Scattering Angle and Energy of Electrons in Xe Atoms

**Figure 3:**
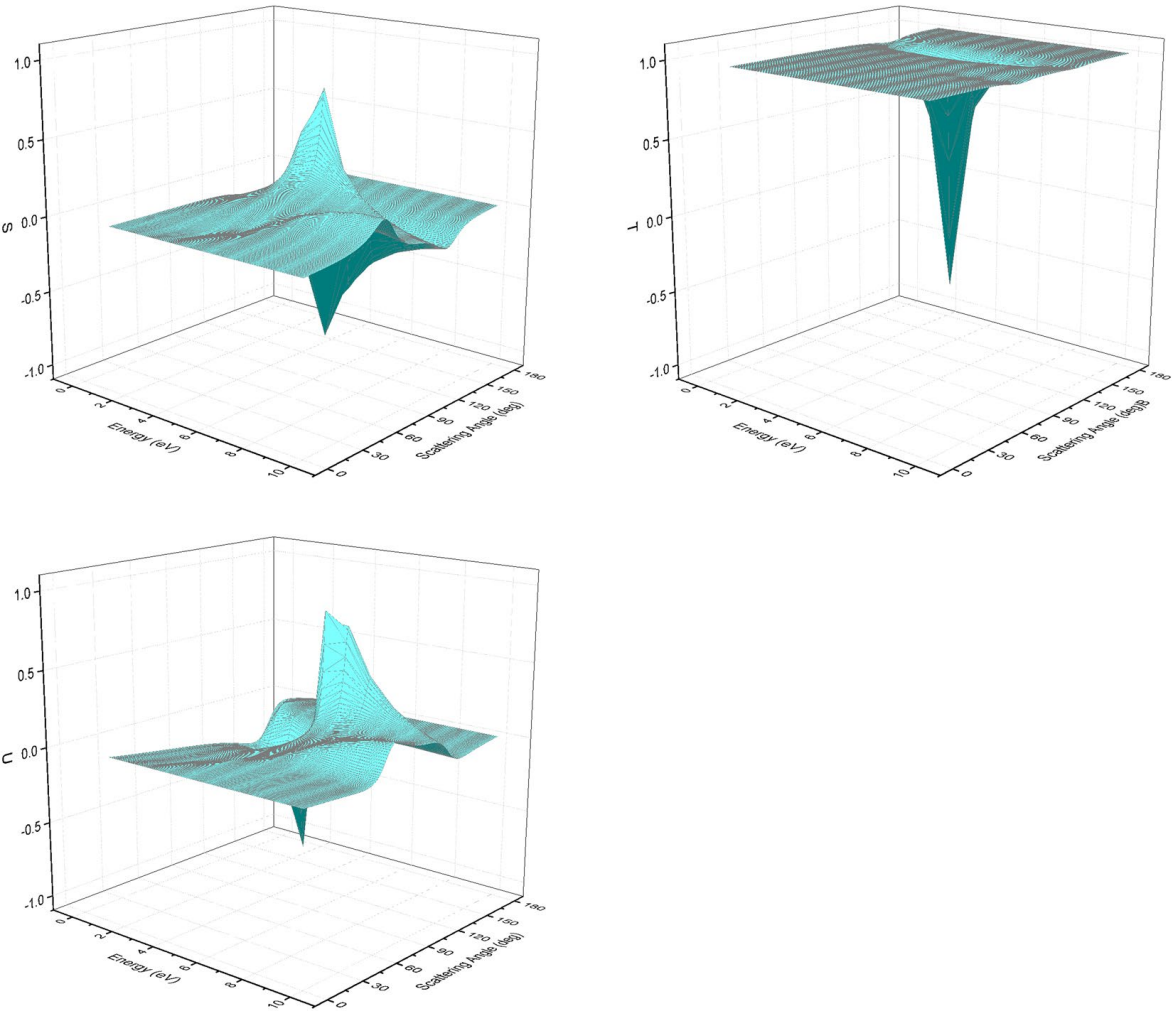
*S*, *T*, and *U* functions of 1.5 eV–10 eV polarized electrons from Xe atoms. POLARe calculations (Cox and Bonham screening function^15^).

The calculated polarization parameters *S*, *T*, and *U* of electrons in Xe atoms provided by POLARe are shown in Fig. 3 as a function of the scattering angle and of the incident electron energy (1.5 eV–10 eV) (Cox and Bonham screening function).

**Figure 4:**
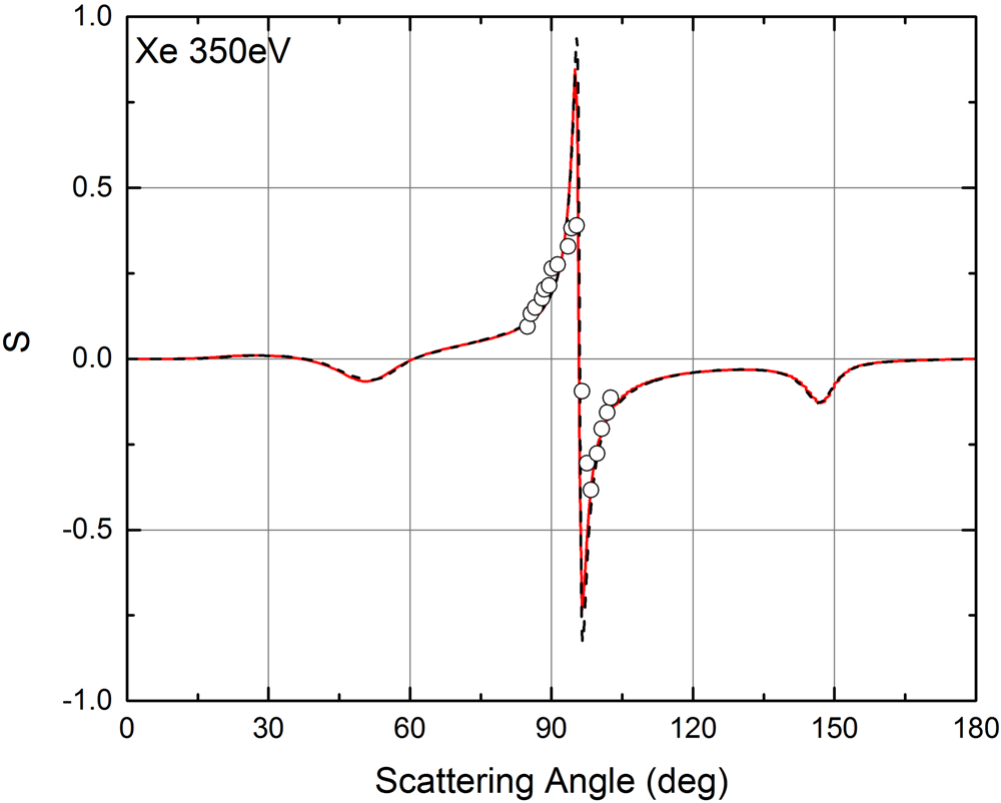
Left-right asymmetry function *S*(*θ*) of 350 eV polarized electrons from Xe atoms. Red solid line: ELSEPA calculations. Dashed black line: POLARe calculations (Cox and Bonham screening function^15^). Symbols: Berger and Kessler experimental data^25^.

**Figure 5:**
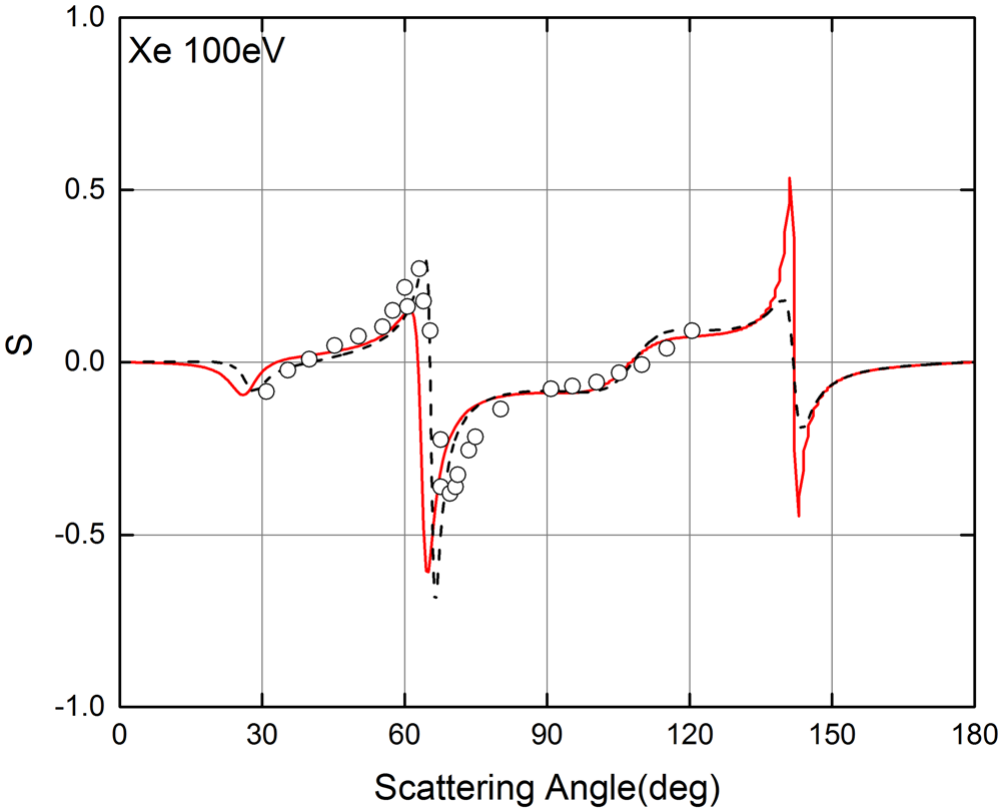
Left-right asymmetry function *S*(*θ*) of 100 eV polarized electrons from Xe atoms. Red solid line: ELSEPA calculations. Dashed black line: POLARe calculations (Cox and Bonham screening function^15^). Symbols: Berger and Kessler experimental data^25^.

**Figure 6:**
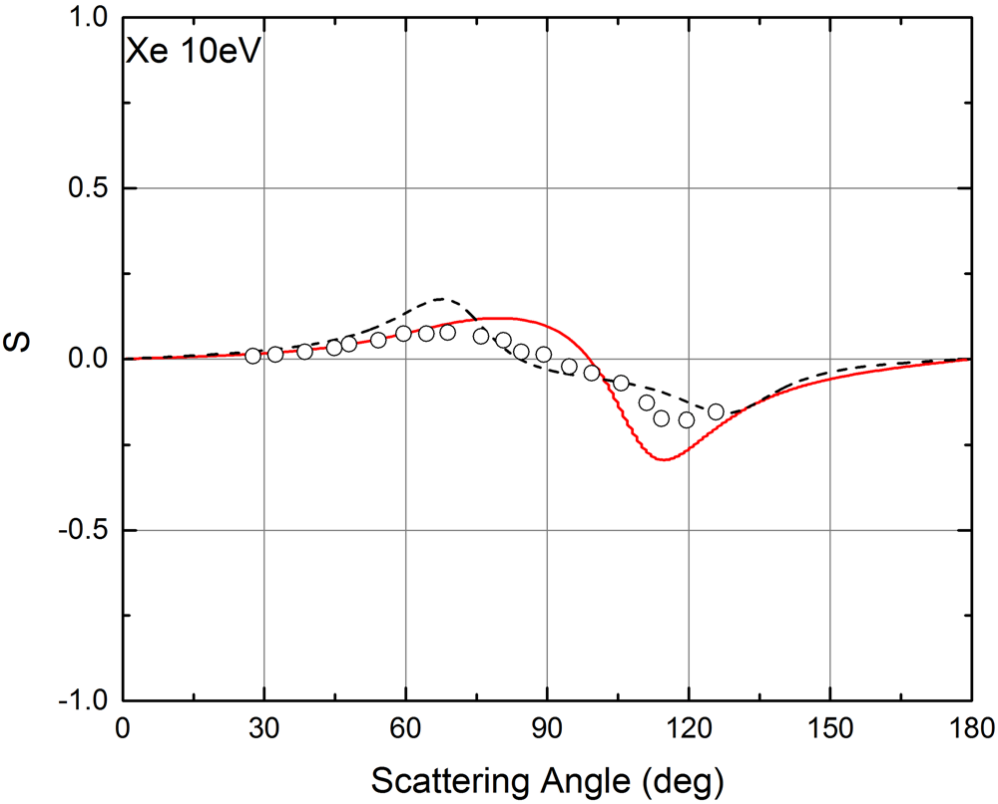
Left-right asymmetry function *S*(*θ*) of 10 eV polarized electrons from Xe atoms. Red solid line: ELSEPA calculations. Dashed black line: POLARe calculations (Cox and Bonham screening function^15^). Symbols: Dümmler *et al*. experimental data^29^.

When the electron energy is higher than 10 eV, also ELSEPA allows to calculate the *S* function. A comparison of the POLARe and of the ELSEPA results concerning the *S* function for energies higher than 10 eV can thus be performed. It is then very useful to investigate the agreement of the results of the two codes with the available experimental data. The comparison between the POLARe and the ELSEPA calculations of the *S* function of Xe for 350 eV electrons is presented in Fig. 4. Also the Berger and Kessler^25^ experimental data are presented in the same figures. The calculations made with the two codes for this electron energy give results practically indistinguishable, and in very good agreement with the experimental data. In Fig. 5 and in Fig. 6 a comparison between the results of the two codes is also presented for the cases of 100 eV and 10 eV electrons in Xe, respectively. In these two cases the calculations provide results showing some differences. In general, even in these cases a reasonable agreement with the experiment by Berger and Kessler^25^ (100 eV) and Dümmler *et al*.^29^ (10 eV) is obtained using both codes. The observed differences between the results of POLARe and ELSEPA can be attributed to the differences in the atomic potentials utilized. The effect of the choice of potential becomes more and more important as the electron energies decrease. It tends to disappear as the electron energy increases above 100 eV.

**Figure 7:**
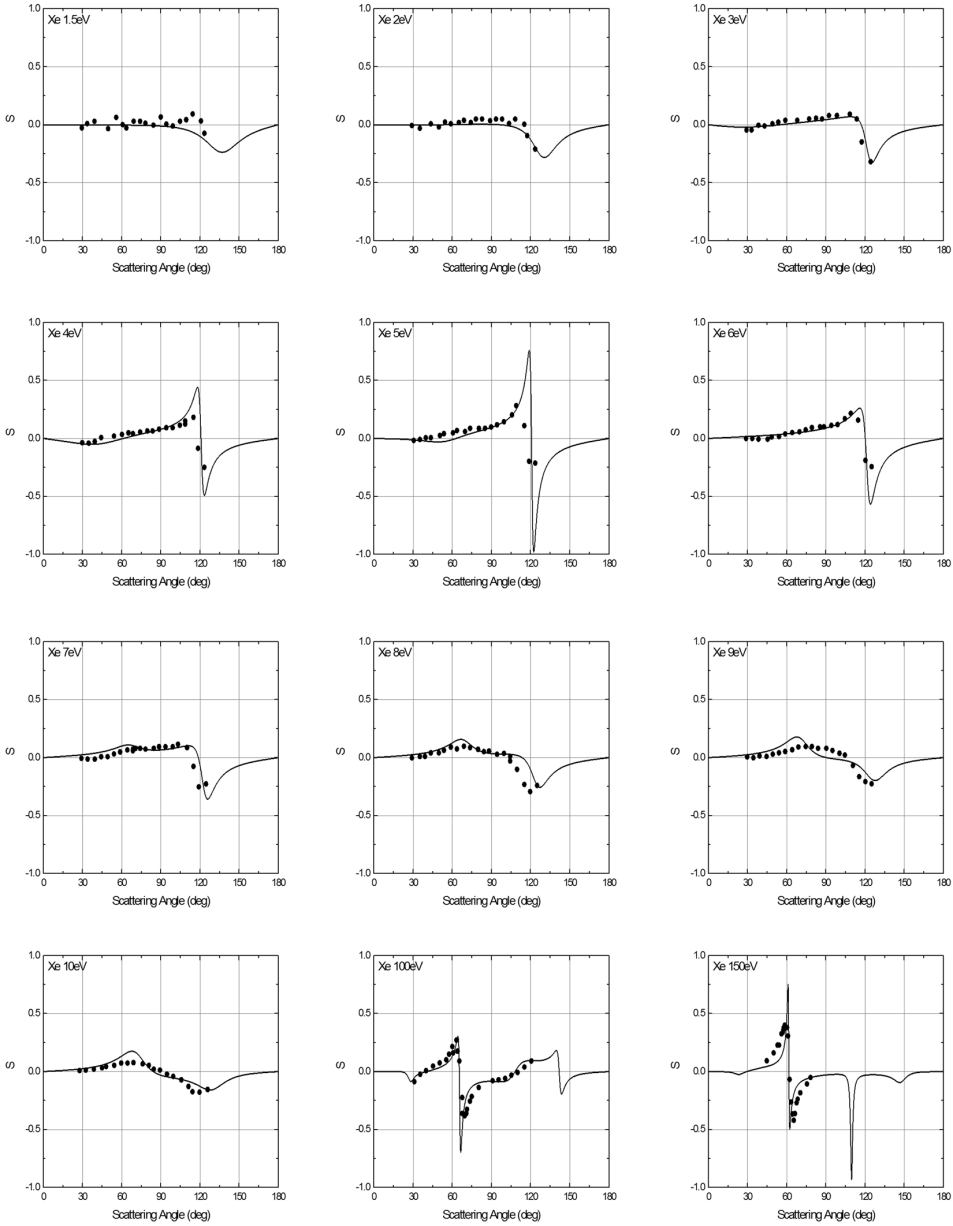
Left-right asymmetry function *S*(*θ*) of 1.5 eV–150 eV polarized electrons from Xe atoms. Solid lines: POLARe calculations (Cox and Bonham screening function^15^). Symbols: Dümmler et al. experimental data^29^ (1.5 eV–10 eV); Berger and Kessler experimental data^25^ (100 eV–150 eV).

The comparison between the POLARe calculations–obtained using the Cox and Bonham screening function–of the *S* function and experimental data concerning 1.5 eV–150 eV electron beams impinging on Xe atoms is presented in Fig. 7.

Basic reasons are at the origin of the observed deviations between experiment and theory, in particular for very low electron kinetic energy (<10 eV). In fact the assumption of a rigid static potential is probably inaccurate when the electron kinetic energy is small. Atomic cloud polarization (note that it has not to be confused with the spin polarization of the electron beam), due to the passage of very slow electrons, is often considered as responsible for the observed discrepancies between theory and experiment. In fact, dynamical effects due to the polarization of the charge cloud of the target atom modify the potential. The induced dipole moment attracts the electrons so that, if the partial wave analysis is based on a rigid static potential, it provides results that can differ from the experimental data. For example, it is known that, neglecting the polarization of the cloud charge, the electron elastic mean free path in solid targets predicted by the partial wave analysis at very low energies is abnormally small. According to Ganachaud and Mokrani^48^ the elastic scattering cross-section obtained with a rigid static potential should be multiplied by a “cut-off” function which has the role to appreciably reduce the elastic effects in solid targets at low energies. According to Salvat *et al*.^40^ the inclusion of the polarization Buckingham potential in the ELSEPA code also improves the accuracy of the calculation of the elastic scattering cross-section of slow electrons.

Thus, on the one hand, the polarization of the charge cloud, not included in the Cox and Bonham potential (which does not depend on the electron kinetic energy), could be the cause of the observed discrepancies between the calculations and the experimental data about the Sherman function. On the other hand, for the case of the presented calculations of the Sherman function, the disagreement between theory and experiment is surprisingly small, even when the electron energy is very low, i.e. from 1.5 eV to 10 eV.

The reasonable agreement we found out between calculations and experimental data for very low energy electron beams can be attributed to the fact that the cloud atomic polarization, induced by low energy electrons, is very small for Xe, as the potential of this atom is reasonably rigid even for a few eV electrons (therefore only slightly affecting the calculation of the Sherman function).

### Exchange Effect

Another possible cause of the observed differences between experiment and theory is related to the exchange effect. In fact, in order to improve the accuracy of the calculation, also the exchange effect should be taken into account when the incident particles are electrons. It is not easy to deal with the exchange effect because, as in the case of bound states, it generates a non-local term in the wave equation^28,49^.

Anyway, when the atomic number is relatively low (*Z* < 40), exchange effect can be described by an approximation proposed by Furness and McCarty^18^ that transforms the non-local term in a local form. The non-relativistic Furness and McCarty formula^18^ is given by

33 $${V}_{{\rm{ex}}}=\frac{1}{2}[E\,-{V}_{{\rm{s}}}]-\frac{1}{2}\sqrt{E-{V}_{{\rm{s}}}+4\pi \rho {e}^{4}{a}_{o}},$$

where *V*_s_ is the atomic potential energy,

34 $${V}_{{\rm{s}}}=-\,\frac{Z{e}^{2}}{r}\,\psi (r)\,,$$

**Figure 8:**
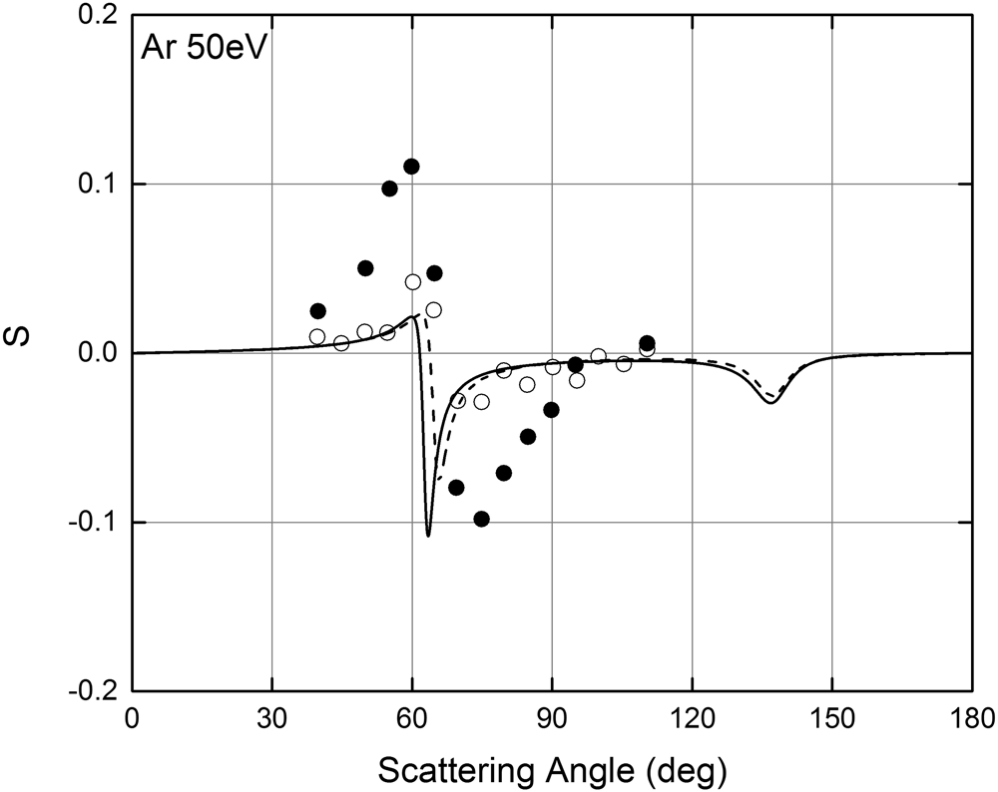
Left-right asymmetry function *S*(*θ*) of 50 eV polarized electrons from Ar atoms. Solid lines: POLARe calculations (Cox and Bonham screening function^15^ without exchange). Dashed line: POLARe calculations (Cox and Bonham screening function^15^ with exchange calculated according to Furness and McCarty^18^). Filled symbols: Schackert experimental data^16^). Empty symbols: Beerlage *et al*. experimental data^21^.

**Figure 9:**
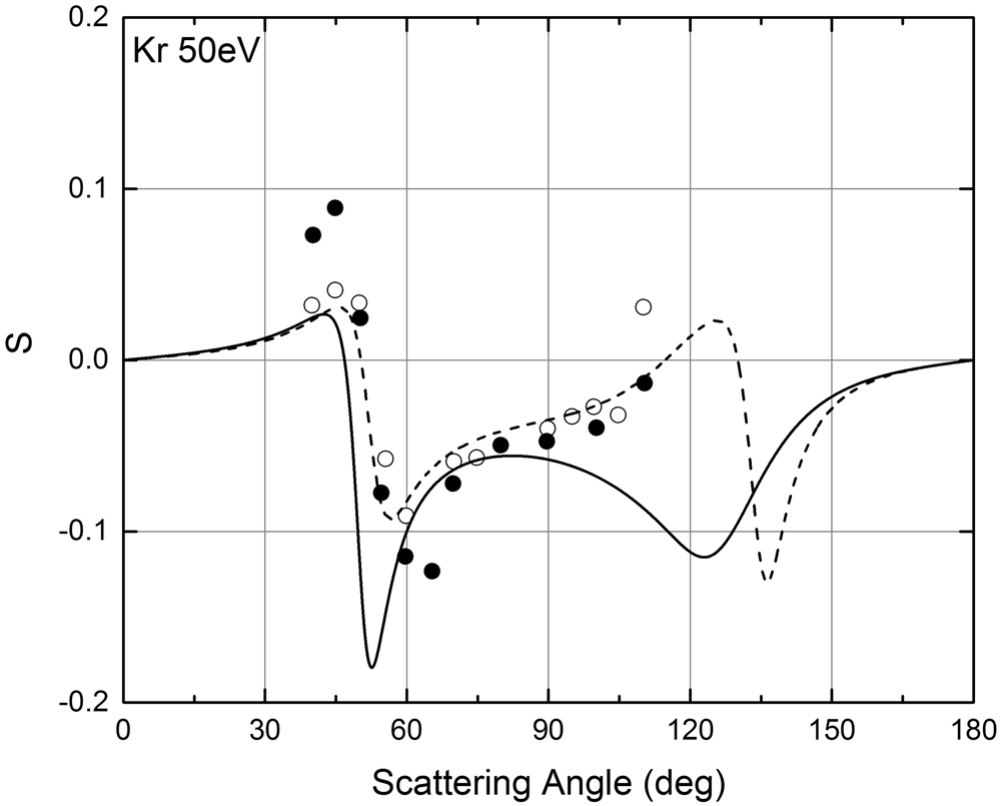
Left-right asymmetry function *S*(*θ*) of 50 eV polarized electrons from Kr atoms. Solid lines: POLARe calculations (Cox and Bonham screening function^15^ without exchange). Dashed line: POLARe calculations (Cox and Bonham screening function^15^ with exchange calculated according to Furness and McCarty^18^). Filled symbols: Schackert experimental data^16^). Empty symbols: Beerlage et al. experimental data^21^.

**Figure 10:**
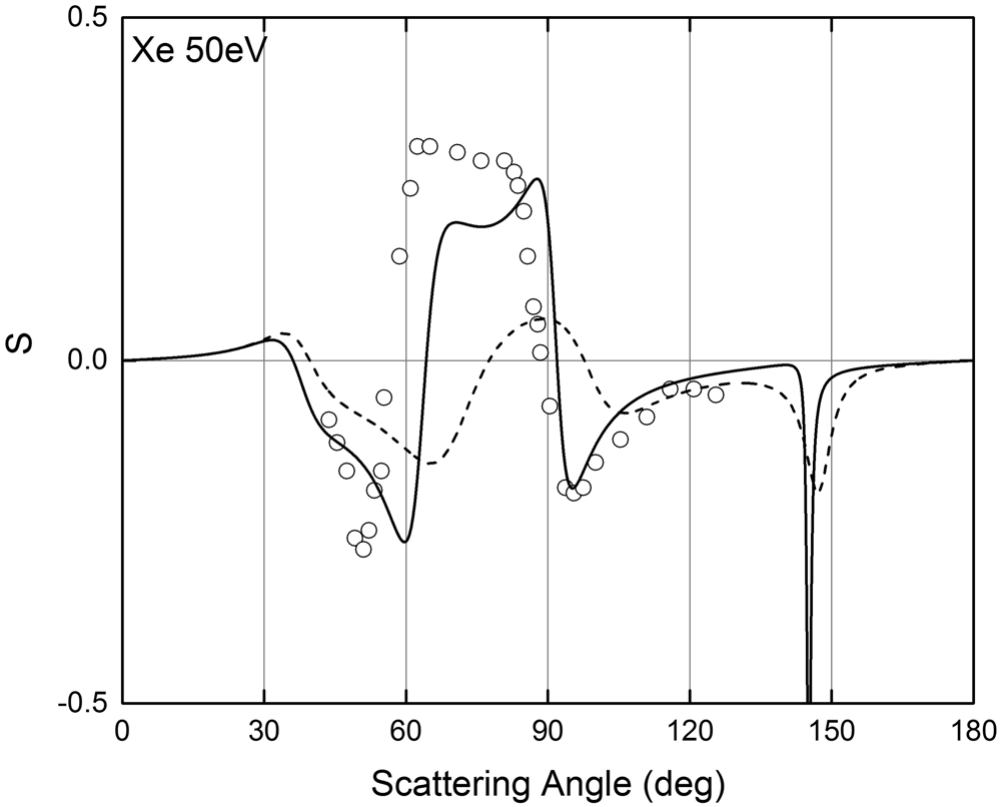
Left-right asymmetry function *S*(*θ*) of 50 eV polarized electrons from Xe atoms. Solid lines: POLARe calculations (Cox and Bonham screening function^15^ without exchange). Dashed line: POLARe calculations (Cox and Bonham screening function^15^ with exchange calculated according to Furness and McCarty^18^). Symbols: Berger and Kessler experimental data^25^.

*ψ*(*r*) is the screening function, (that can be calculated using the Cox and Bonham best fit parameters [see Eq. (31)]), and *ρ* is the electron density [see Eq. (32)]. Figures 8–10 show the comparison of the POLARe calculations with the experimental data of Schackert^16^, Beerlage *et al*.^21^, and Berger and Kessler^25^ of 50 eV electrons in Ar, Kr, and Xe, respectively. In order to study the effect of the use of the Furness and McCarty non-relativistic approximation on the calculation of the spin-polarization, in Figs 8–10 the POLARe calculations were presented with and without exchange.

The comparison shows that, as expected, the inclusion of the exchange using a non-relativistic approximation provides a better agreement with the experimental data when the atomic number is relatively low (Ar, *Z* = 18 and Kr, *Z* = 36). For the case of Xe (Z = 54), on the other hand, the inclusion of the Furness and McCarty model in the calculation of the potential worsens the agreement. A possible explanation of this behavior is related to the non-relativistic arguments used to obtain the Furness and McCarty formula.

In conclusion, on the basis of the present observations, the inclusion of the exchange effect by the use of the Furness and McCarty non-relativistic approximation is recommended, but only when the atomic number of the target is relatively low.

## Conclusion

The density-matrix formalism was used to investigate spin-polarization phenomena in the electron-atom elastic scattering. The POLARe code, a computer program written to calculate the spin-polarization parameters characterizing the elastic scattering of electrons with atomic targets, was described. Selected results of the program concerning Ar, Kr, and Xe atoms were presented. We found out a reasonable agreement with experimental data even when the incident electron kinetic energy was very low (smaller than 10 eV).

## Electronic supplementary material

Supplementary Information
