# Publisher Correction: Polarized electron beams elastically scattered by atoms as a tool for testing fundamental predictions of quantum mechanics

**DOI:** 10.1038/s41598-018-25258-2

**Published:** 2018-04-26

**Authors:** Maurizio Dapor

**Affiliations:** 1 European Centre for Theoretical Studies in Nuclear Physics and Related Areas (ECT*-FBK), Trento, 38123 Italy; 2 Trento Institute for Fundamental Physics and Applications (TIFPA-INFN), Trento, 38123 Italy

Correction to: *Scientific Reports* 10.1038/s41598-018-23660-4, published online 29 March 2018

The PDF version of this Article previously contained an incorrect link to the Supplementary Information.

Additionally, this Article contained an error in the legend of Figure 8 where,

“Solid lines: POLARe calculations (Cox and Bonham screening function^15^ with exchange).”

now reads:

“Solid lines: POLARe calculations (Cox and Bonham screening function^15^ without exchange).”

These errors have now been corrected in the HTML and PDF versions of this Article.

