# Supplementary material for: Polarized electron beams elastically scattered by atoms as a tool for testing fundamental predictions of quantum mechanics
Source: Sci Rep. 2018 Mar 29;8:5370. doi: 10.1038/s41598-018-23660-4 (PMC5876396; doi:10.1038/s41598-018-23660-4)
Supplement: Supplementary file 1 — Supplementary Information [file 41598_2018_23660_MOESM1_ESM.pdf]

Supplementary information
Polarized electron beams elastically scattered by atoms as a tool for testing fundamental predictions of quantum mechanics

Maurizio Dapor[1,2,*]

[1]European Centre for Theoretical Studies
in Nuclear Physics and Related Areas
(ECT*-FBK)
Trento, 38123, Italy

[2]Trento Institute
for Fundamental Physics and Applications
(TIFPA-INFN)
Trento, 38123, Italy

March 21, 2018

Density Matrix Formalism for Spin Polarization

In this section we present the density matrix formalism for spin polarization in a very simple, pedagogical form. Even if this formalism is well known (see, for example, Refs. [1–3]) it seems useful to summarize it, in the present context, for the convenience of the reader.

An electron beam is constituted by n sub-systems each of which is in a pure state. Thus the spin orientation is known through a probability distribution. Let us indicate with $|a\rangle$ the normalized state vectors representing the pure states and with $|i\rangle$ a complete set of of orthonormal eigenvectors so that

$$|a\rangle = \sum_i c_i |i\rangle \tag{1}$$

where

$$c_i = \langle i|a\rangle \,. \tag{2}$$

Let be p_a the probability of obtaining the pure state $|a\rangle$. The average value of an operator A is

$$\langle A\rangle \;=\; \sum_{a=1}^{n} p_a\,\langle a|A|a\rangle\,. \tag{3}$$

The density operator $\hat{\rho}$ is defined as

$$\hat{\rho} \;=\; \sum_{a=1}^{n} p_a\,|a\rangle\langle a|\,. \tag{4}$$

The calculation of the matrix elements $\hat{\rho}_{ij}$ of the density matrix,

$$\hat{\rho}_{ij} \;=\; \langle i|\hat{\rho}|j\rangle \;=\; \sum_{a=1}^{n} p_a\langle i|a\rangle\langle a|j\rangle = \sum_{a=1}^{n} p_a c_i c_j^*\,, \tag{5}$$

allows us to demonstrate that the average value of any operator A is given by the trace of $\hat{\rho}A$:

$$\langle A\rangle \;=\; \sum_{a=1}^{n}\sum_{j}\sum_{i} p_a\langle a|j\rangle\langle j|A|i\rangle\langle i|a\rangle \;=\; \sum_{i}\sum_{j}\langle i|\hat{\rho}|j\rangle\,\langle j|A|i\rangle\,. \tag{6}$$

Since the set of orthonormal eigenvectors $|j\rangle$ is complete, then

$$\sum_{j}|j\rangle\langle j| \;=\; I\,, \tag{7}$$

where I is the identity operator. As a consequence

$$\langle A\rangle \;=\sum_{i}\langle i|\hat{\rho}A|i\rangle \;=\; \mathrm{T}r(\hat{\rho}A)\,. \tag{8}$$

Let us now consider the spin space. A complete set of 2×2 Hermitean matrices is given by the Pauli matrices σ_k and the identity matrix I. As a consequence the density matrix can be expressed on this basis as

$$\hat{\rho} \;=\; \alpha_0\,I \;+\sum_{k=1}^{3}\alpha_k\,\sigma_k\,, \tag{9}$$

where α_k, with $k = 0,\,1,\,2,\,3$, are real coefficients. It is quite easy to find that the value of α_0 is $1/2$. In fact, let us first observe that, since $|a\rangle$ are normalized and $\sum_{a=1}^{n} p_a \;=\; 1$, the trace of the density matrix is 1. In fact

$$\mathrm{T}r(\hat{\rho}) \;=\; \mathrm{T}r(\hat{\rho}\,I) \;=\langle I\rangle \;=\; \sum_{a=1}^{n} p_a\,\langle a|I|a\rangle \;=\; \sum_{a=1}^{n} p_a\,\langle a|a\rangle \;=\; 1\,. \tag{10}$$

Furthermore it is easy to see that

$$\mathrm{T}r(\sigma_k) \,=\, 0\,, \tag{11}$$

for $k = 1, 2, 3$, so that

$$1 \,=\, \mathrm{T}r(\hat{\rho}) \,=\, \alpha_0\,\mathrm{T}r(I) \,=\, 2\,\alpha_0\,. \tag{12}$$

The values of α_k, for $k = 1, 2, 3$, can be obtained observing that, since (i) $\sigma_k^2 = I$, $\mathrm{T}r(\sigma_k^2) = 2$ and, (ii) when $k \neq l$, $\mathrm{T}r(\sigma_k\,\sigma_l) = -\mathrm{T}r(\sigma_k\,\sigma_l) = 0\,,$

$$\mathrm{T}r(\sigma_k\,\sigma_l) \,=\, 2\,\delta_{kl}, \tag{13}$$

so that

$$\langle\sigma_k\rangle \,=\, \mathrm{T}r(\hat{\rho}\,\sigma_k) \,=\, 2\,\alpha_k\,. \tag{14}$$

Let us now define the components of the polarization vector $\mathbf{P}$, P_k ($k = 1, 2, 3$), as the average values of the Pauli matrices:

$$P_k \,\equiv\, \langle\sigma_k\rangle\,. \tag{15}$$

The density matrix can thus be expressed as a function of the polarization as follows:

$$\hat{\rho} \,=\, \frac{1}{2}\left(I \,+\, \sum_k \sigma_k\,P_k\right)\,. \tag{16}$$

Selecting the z axis in the direction of the polarization, so that $P_1 = 0$, $P_2 = 0$, and $P_3 = P$,

$$\hat{\rho} \,=\, \frac{1}{2}\,(I \,+\, \sigma_3\,P\,)\,. \tag{17}$$

In this case, using the usual representation of the Pauli matrices, the diagonal elements of the density matrix are given by

$$\hat{\rho}_{11} \,=\, \frac{1+P}{2}\,, \tag{18}$$

$$\hat{\rho}_{22} \,=\, \frac{1-P}{2}\,, \tag{19}$$

while the off-diagonal elements are given by $\hat{\rho}_{12} = \hat{\rho}_{21} = 0$.

The diagonal element $\hat{\rho}_{11}$ of the density matrix represent the probability that an electron of the beam is found in the state 1 (spin up). Similarly $\hat{\rho}_{22}$ is the probability that an electron of the beam is found in the state 2 (spin down).

Indicating with u the number of electrons of the beam with spin up and with d the number of electrons of the beam with spin down we can write

$$\frac{1+P}{2} \,=\, \frac{u}{u+d}\,, \tag{20}$$

so that

$$P = \frac{u - d}{u + d} \,. \tag{21}$$

Also note that

$$\hat{\rho} = (1 - P)\,\hat{\mu} + P\,\hat{\nu} \tag{22}$$

where the density matrices $\hat{\mu}$ and $\hat{\nu}$ are diagonal with $\hat{\mu}_{11} = \hat{\mu}_{22} = 1/2$, $\hat{\nu}_{11} = 1$, $\hat{\nu}_{22} = 0$. The density matrix $\hat{\mu}$ corresponds to a completely unpolarized beam, as the probability to find in the beam an electron with spin up is identical with the probability to find in the beam an electron with spin down. On the other hand the density matrix $\hat{\nu}$ corresponds to a pure state in which all the electrons of the beam have spin up (the beam in this case is totally polarized).

References

[1] Kessler, J. *Polarized Electrons* (Springer-Verlag, Berlin, 1985).

[2] Farago, P.S. Electron spin polarization. *Rep. Prog. Phys.* **34**, 1055-1124 (1971).

[3] Dapor, M. *Electron-Beam Interactions with Solids: Application of the Monte Carlo Method to Electron Scattering Problems* (Springer Tracts in Modern Physics 186, Springer 2003).
